# Developing National Information Systems to Monitor COVID-19 Vaccination: A Global Observational Study

**DOI:** 10.2196/62657

**Published:** 2024-10-25

**Authors:** Donald J Brooks, Carolyn Inae Kim, Franck Fortune Mboussou, M Carolina Danovaro-Holliday

**Affiliations:** 1 Department of Immunization, Vaccines and Biologicals, World Health Organization, Avenue Appia 20, Geneva, 1211, Switzerland, 41 0795090626; 2 Regional Office for Africa, World Health Organization, Brazzaville, Congo

**Keywords:** COVID-19, COVID-19 vaccine, immunization information system, vaccination monitoring, vaccine, monitoring and evaluation

## Abstract

**Background:**

Strong information systems are essential for safe and effective immunization programs. The COVID-19 vaccine rollout presented all immunization information systems (IIS) with challenging demands—requiring in-depth vaccine implementation data at all health system levels in real time. The system development approaches taken by countries were heterogeneous, with some countries opting to adapt existing systems and others implementing new ones.

**Objective:**

Using data reported by Member States to the World Health Organization (WHO), we aim to develop a global understanding of (1) the types of IIS used to monitor COVID-19 vaccination implemented in 2021 and (2) the approaches taken by countries to develop these systems.

**Methods:**

We conducted a descriptive analysis of data reported through a supplemental questionnaire of the WHO/United Nations Children's Emergency Fund (UNICEF) Joint Reporting Form on Immunization, collecting data for 2021 on (1) the use of and developmental approaches taken for 7 IIS functions (appointments, aggregate reporting, individual-level reporting, reminders, home-based records, safety surveillance, and stock management), and (2) modifications needed for digital health frameworks to permit COVID-19 vaccination monitoring.

**Results:**

In total, 188 of 194 WHO Member States responded to the supplemental questionnaire, with 155 reporting on the IIS-related questions. Among those reporting, for each of the 7 IIS functions explored, greater than 85% of responding countries reported that the system was in place for COVID-19 vaccines. Among responding countries, “aggregate reporting system” was the system most frequently reported as being in place (n=116, 98.3%), while “reminder system” was the least (n=77, 89%). Among the countries reporting using a system, whether an existing system was adapted for COVID-19 vaccines or a new one was developed varied by system. Additionally, two-thirds (n=127, 67.6%) of countries reported establishing at least one new system, ranging from 72% (n=42) in high-income countries (HICs) to 62% (n=16) in low-income countries. Concurrently, 55.3% (n=104) of countries reported adapting at least one system already in place for COVID-19 vaccines, with 62% (n=36) of HICs reporting this compared to about 53% for other income groups. Of those reporting developing new systems, for each of the systems explored, more than 85% of countries reported that they intended to keep new systems specific to COVID-19 vaccines. Further, 147 of the 188 (78.2%) Member States responding to the supplemental questionnaire responded to the digital health frameworks question. Lastly, 31% (n=46) of responding countries reported needing to adapt them for COVID-19 vaccination systems. HICs had a higher percentage.

**Conclusions:**

Nearly all countries have adapted existing or developed new IIS to monitor COVID-19 vaccination. The approaches varied, notably by income group. Reflection is needed on how to sustain the investments made in IIS during the pandemic. Continued support for IIS is critical, given their essential role in program monitoring and performance.

## Introduction

Strong information systems are essential for safe and effective immunization programs. Information systems play key roles across the vaccine implementation value chain, implicated in stock management, personal records, and operational, public health, and safety monitoring. Armed with accurate, timely “fit-for-purpose” data from these systems, health officials can monitor vaccination program process indicators and progress against established goals, ensuring they are equitably and safely reaching targeted populations with recommended vaccines [1]. Importantly, robust information systems allow health officials to identify and correct obstacles preventing smooth program implementation, whether these obstacles be related to supply, service delivery strategy, or other factors [2].

As the largest and fastest vaccine implementation in history, the COVID-19 vaccine rollout presented all immunization information systems (IIS) with challenging, new demands. Within the span of 6 months, from December 2020 to June 2021, nearly every country in the world introduced a COVID-19 vaccine [3]. Following introduction, most countries aimed to vaccinate 70% of their total populations, prioritizing risk groups, as quickly as possible, striving to reach aspirational global goals [4]. This required stock, administrative, and safety monitoring systems capable of handling quantities of doses, patients, and safety signals’ orders of magnitude larger than those under routine immunization programs. Given the urgency, too, these data were needed for decision-making in real time at all levels of the health system, further breaking from routine programs with their often-monthly reporting cadences.

As a result, all countries needed to rapidly adapt existing information systems and governance frameworks, or develop new ones, to meet these data needs. Reports from early in the rollout highlighted that countries were using a range of strategies to ensure collection and analysis of necessary COVID-19 vaccination-related data. Strategy choice was strongly influenced by baseline IIS strength and maturity, which vary widely between countries [2]. Some countries, including Denmark and Chile, leveraged and expanded existing systems and frameworks to include COVID-19 vaccination [5,6]. For others, especially for those not having previously introduced an adult-targeted vaccine, COVID-19 vaccines served as a catalyst for the adoption of new systems. In many cases, these new systems use digital technologies, accelerating the digital transformation of health systems [7]. This was the case in Nigeria and the United Kingdom, among others [8,9].

Given the observed heterogeneity in the systems used to monitor COVID-19 vaccination, it is important to identify and understand patterns in the approaches taken by countries. Such a landscaping analysis will enable, guide, and promote subsequent assessment of these approaches to determine what worked and what did not. This understanding is critical to informing new and ongoing IIS strengthening activities and investments. This is true not only for sustaining capacity gains made during COVID-19, but also for ensuring that future investments are preparing monitoring systems for the immunization programs of the future, including preparedness for future epidemic or pandemic disease outbreaks [10].

To develop a global understanding of the types of IIS implemented in 2021 to monitor COVID-19 vaccination, the World Health Organization (WHO) and UNICEF (United Nations Children's Emergency Fund) developed and administered a questionnaire on the topic to their Member States. The questionnaire was administered through the annual WHO/UNICEF Joint Reporting Form on Immunization (JRF) data collection process [11]. We describe the results of that questionnaire and discuss how IIS are rapidly evolving as a result of the COVID-19 pandemic and the rollout of COVID-19 vaccines.

## Methods

### Data Collection

Data were reported to WHO and UNICEF through a supplemental questionnaire of the annual JRF, collecting data for 2021. The JRF for 2021 was sent to Member States (“countries”) in March 2022. The supplemental questionnaire was designed to collect information on routine immunization disruption and recovery in the context of the COVID-19 pandemic.

To assess the information systems used to manage COVID-19 vaccines, the supplemental questionnaire included the following question: “In 2021, to manage COVID-19 vaccines, routine immunization, and vaccination monitoring (in COVID-19 context), which of the following systems were used?” The question covered seven IIS functions: (1) vaccination appointments, determination eligibility and scheduling (“appointment system”), (2) aggregate system for recording and reporting vaccinations or e-Health Management Information System (“aggregate reporting system”), (3) individual registration and reporting (electronic immunization registry, “EIR”), (4) system to send recall or remind (reminder system), (5) electronic home-based record (“eHBR”), (6) adverse event following immunization surveillance system (“safety system”), and (7) supply chain management (“stock system”; Multimedia Appendix 1). For each of the seven systems, countries could indicate that they: (1) adapted a system that was already in place for routine immunization for COVID-19, (2) developed a new system for COVID-19 vaccines only, (3) developed a new system for COVID-19 vaccines which is being or will be expanded to other routine vaccines, or (4) if an electronic (“digital”) system was not in place. Multiple options could be selected for each system to capture the possibility that multiple types of the same system were used or if the types of systems used varied between health system levels. Descriptions of each system were provided via tooltips in the questionnaire (Multimedia Appendix 2).

To assess digital health framework in countries, countries were asked, “In 2021, were there any adaptations (legislation, guidelines for interoperability, partnering with private providers, etc) needed for the governance framework used for digital health in relation to the systems used for COVID-19 vaccination and/or routine immunization in the COVID-19 context?” Countries could indicate “yes” or “no” or leave it blank.

### Data Cleaning and Analysis

Questionnaire response data were analyzed using RStudio statistical software (version 2022.02.3, Posit, PBC). Data were stratified by WHO region, World Bank income classification (2023 revision), Gavi-eligibility status as of 2024, population size (United Nations population estimates of surviving infants, 2022 revision), and COVID-19 vaccination coverage (WHO COVID-19 vaccine administration data system).

A country was considered as having responded to the supplemental questionnaire if it had completed any question, not limited to the IIS-related questions. A country was considered as having responded to the IIS-related questions if it had completed any IIS-related question. For the IIS questions, countries were considered as having a system in place if they responded “yes” to any of the following three options: (1) system that was already in place and was adapted to COVID-19, (2) a new system was developed for COVID-19 vaccines only, or (3) a new system developed for COVID-19 vaccines and was, is being, or will be expanded to other routine vaccines. If a country reported both not having a system in place and that a system was in place (either existing or new system), the country was considered as having a system in place. Countries were considered both as already having a system in place and as having developed a new system if they indicated both options for a specific system. Similarly, countries were considered both as having an intention and not having an intention to expand new systems to other routine vaccines if they selected both options for a specific system. Those that did not provide a response to the relevant question were excluded from further analysis.

### Ethical Considerations

No individual-level data were used in this study. Only country-level data, officially reported by Ministries of Health to WHO and UNICEF, were used. As a result, ethical approval from a research board was not considered relevant.

## Results

### Reporting Overview

Of the 194 WHO Member States to whom it was sent, 188 submitted the supplemental questionnaire, as of August 10, 2023. Reporting was consistent across WHO regions and income groups, with response rates ranging from 89% to 100% and from 94% to 100%, respectively (Table 1). Reporting countries contained approximately 98% of the global population.

**Table 1.** WHO^a^ Member States (1) submitting the supplemental questionnaire of the 2021 JRF^b^ on routine immunization disruption and recovery in the context of the COVID-19 pandemic and (2) responding to at least one of the IIS^c^-related questions, stratified by WHO region and income group.

		WHO Member States submitting the supplemental questionnaire, n/N (%)	WHO Member States responding to at least one IIS-related question, n/N (%)
**WHO region**			
	AFR^d^	46/47 (98)	39/47 (83)
	AMR^e^	35/35 (100)	27/35 (77)
	EMR^f^	19/20 (95)	12/20 (60)
	EUR^g^	53/53 (100)	40/53 (75)
	SEAR^h^	11/11 (100)	10/11 (91)
	WPR^i^	24/27 (89)	18/27 (67)
**World Bank income classification** ^**j**^			
	Low	26/26 (100)	21/26 (81)
	Low-middle	51/54 (94)	42/54 (78)
	Upper-middle	51/52 (98)	38/52 (73)
	High	58/59 (98)	44/59 (75)

a WHO: World Health Organization.

b JFR: World Health Organization/United Nations Children's Emergency Fund Joint Reporting Form on Immunization.

c IIS: immunization information systems.

d AFR: WHO African Region.

e AMR: WHO Region of the Americas.

f EMR: WHO Eastern Mediterranean Region.

g EUR: WHO European Region.

h SEAR: WHO South-East Asia Region.

i WPR: WHO Western Pacific Region.

j Based on the 2023 revision of the World Bank income classification. Two countries not classified by the World Bank were excluded (Niue and Venezuela).

### Systems Used to Manage COVID-19 Vaccination

Response completeness varied across the 7 IIS functions explored, as not all countries responding to the supplemental questionnaire overall answered each IIS-related question. The response rate was highest for “safety system” with 66.5% (n=125) of reporting countries responding to that question. “eHBR” featured the lowest response rate with 42% (n=79; Table 2). Response completeness for the remaining IIS functions were the following: 66% (n=124) for “appointment system,” 62.8% (n=118) for “aggregate reporting system,” 61.7% (n=116) for “EIR,” 53.7% (n=101) for “stock system,” and 46% (n=87) for “reminder system.” Further, 27% (n=51) of countries that submitted the supplemental questionnaire provided a response for all 7 systems explored, and 24% (n=46) did not provide a response for any of the 7 systems.

**Table 2.** Proportion of reporting WHO^a^ Member States providing a response for each of the 7 immunization information system functions under study via the supplemental questionnaire.

Immunization information system function	Proportion of reporting WHO Member States, n (%)
Stock system	101 (54)
EIR^b^	116 (62)
eHBR^c^	42 (79)
Aggregate reporting system	118 (63)
Safety system	125 (66)
Appointment system	124 (66)
Reminder system	87 (46)

a WHO: World Health Organization.

b EIR: electronic immunization registry.

c eHBR: electronic home-based record.

### Analysis by System

Worldwide, for each of the 7 systems explored, nearly all responding countries reported having the system in place. Among responding countries, “aggregate reporting system” was the system most frequently reported as being in place (n=116, 98.3%), while “reminder system” was the least (n=77, 89%; Table 3). “Reminder system” was the only system for which less than 90% of responding countries reported using that system. Of the 51 countries that provided a response for all 7 systems, 80% (n=41) responded as having all 7 systems in place. None of the 51 responded as not having any of the 7 systems.

**Table 3.** Breakdown of questionnaire responses on IIS^a^ function use, development approach, and intent to expand the newly developed system, for and as relating to COVID-19 vaccination.

Variable		Supply system (n=101), n (%)	EIR^b^ (n=116), n (%)	eHBR^c^ (n=79), n (%)	Aggregate reporting system (n=118), n (%)	Safety system (n=125), n (%)	Appointment system (n=124), n (%)	Reminder system (n=87), n (%)
**Use status**								
	System in use	98 (97)	110 (94.8)	71 (90)	116 (98.3)	116 (92.8)	116 (93.5)	77 (89)
**Development approach**								
	System already in place adapted	66 (65)	42 (36)	25 (32)	64 (54)	76 (61)	41 (33)	26 (30)
	New system developed	52 (51)	86 (74)	54 (68)	87 (74)	63 (50)	101 (81.5)	62 (71)
**Intent to expand newly developed systems**								
	Developed only for COVID-19	45 (87)	82 (95)	49 (91)	76 (87)	60 (95)	93 (92)	58 (94)
	Developed for COVID-19 and is or will be expanded to other vaccines	16 (31)	32 (37)	22 (41)	35 (40)	33 (52)	32 (32)	23 (37)

a IIS: immunization information systems.

b EIR: electronic immunization registry.

c eHBR: electronic home-based record.

**Figure 1.**
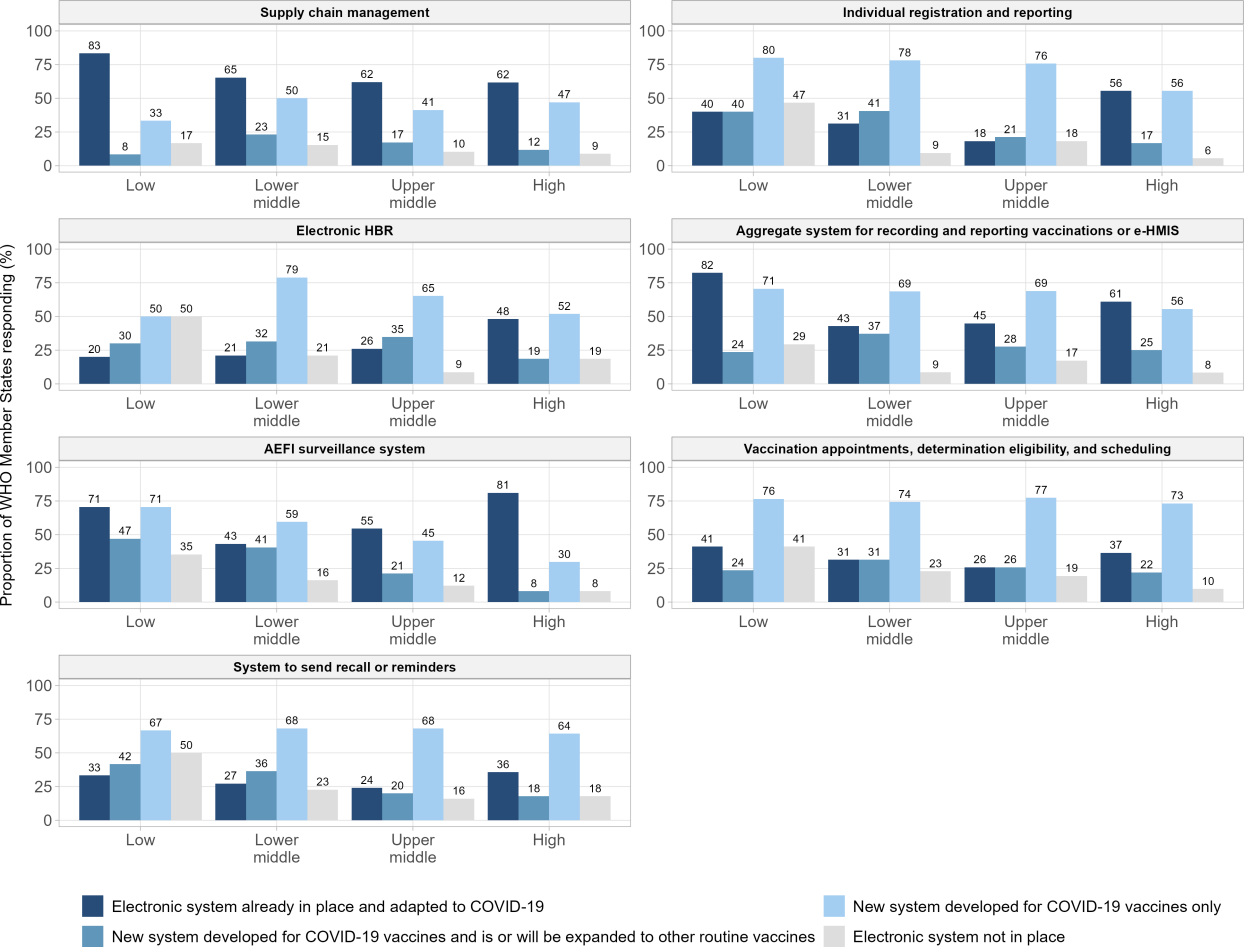
Breakdown of responses on systems in place to manage COVID-19 vaccines and routine immunization vaccination monitoring (in the COVID-19 context) by income group. AEFI: adverse event following immunization; e-HMIS: electronic Health Information Management System; HBR: home-based record; WHO: World Health Organization.

Among the countries reporting using a system, whether an existing system was adapted for COVID-19 vaccines or a new one was developed varied by system. Across all systems, greater than 50% of responding counties reported developing a new system specific to COVID-19 vaccines at some level of the health system. In particular, for each “EIR,” “aggregate reporting system,” and “appointment system,” greater than two-thirds of responding countries reported developing new systems. Adaptation of existing systems was most frequently reported for “stock system” and “safety system.” In both of these cases, a greater percentage of countries reported adapting systems than did those reporting establishing new ones: 65% to 51% and 61% to 50%, respectively (Figure 1). “eHBR,” “appointment system,” and “EIR” were least frequently reported as being adapted (Figure 1). Overall, 67.6% (n=127) reported establishing at least one new system, with 58.5% (n=110) reporting establishing at least two new systems.

Of those reporting developing new systems for COVID-19 vaccines, for each of the 7 systems explored, more than 85% of countries reported that they intended to keep new systems specific to COVID-19 vaccines. The systems most frequently reported as having been kept specific to COVID-19 vaccines were “EIR” and “safety system,” each with 95% of reporting countries indicating this option. The percentage of countries reporting having expanded a new system to other vaccines or intending to do so generally remained around 35% across systems, save for “eHBR,” “aggregate reporting system,” and “safety system,” each having a figure greater than 40% (Table 3). Of the countries implementing at least one new system across any of the 7 systems, 96.9% (n=123) reported that they intended to keep at least one new system specific to COVID-19 vaccines, while 47% (n=60) reported that they had or intended to expand at least one new COVID-19 vaccine system to other vaccines.

### Analysis by System, Stratified by Income Group and WHO Region

Across income groups, the most common approach taken varied by system under study. Rankings of the most common approach taken for “stock management,” “eHBR,” and “reminder system” remain largely consistent across income groups (Figure 1). There is greater variation, however, between income groups for the remaining systems and in particular for “safety system.” For “safety system,” we observe that upper-middle and high-income countries (HICs) were more likely to have electronic systems in place that were subsequently adapted for COVID-19 vaccines, whereas lower-middle and low-income countries (LICs) were more likely to establish new systems (Figure 1). Interestingly, for “aggregate reporting system,” both LICs and HICs more frequently adapted existing systems, as compared with middle income countries, which favored standing up new systems (Figure 1).

Across WHO regions, the ranking of most common approach varies considerably by system under study. “Stock system” and “appointment system” did remain consistent across regions, however (Multimedia Appendix 3).

### Digital Health Frameworks During COVID-19

In total, 147 of the 188 reporting Member States (78.2%) that submitted the supplemental questionnaire responded to the question on digital health frameworks. Overall, 31% (n=46) of responding Member States reported needing adaptations to their digital health governance frameworks in relation to systems used for COVID-19 vaccination. Across WHO regions, the proportion needing adaptations varied from 40% (n=4) in the South-East Asian Region to 22% (n=6) in the Region of the Americas. When looking across income groups, a higher percentage of HICs needed adaptations than other income groups (Table 4).

**Table 4.** Overview of need for adaptation to digital health governance frameworks in relation to systems used for COVID-19 vaccination, at the global level and by country grouping.

Country grouping		Countries responding, n	Countries responding “yes” (as % of those responding), n (%)
Overall		147	46 (31)
**WHO^a^ region**			
	AFR^b^	37	11 (30)
	AMR^c^	27	6 (22)
	EMR^d^	13	4 (31)
	EUR^e^	43	16 (37)
	SEAR^f^	10	4 (40)
	WPR^g^	17	5 (29)
**Income group**			
	Low	19	4 (21)
	Lower-middle	41	10 (24)
	Upper-middle	40	13 (33)
	High	46	19 (41)

a WHO: World Health Organization.

b AFR: WHO African Region.

c AMR: WHO Region of the Americas.

d EMR: WHO Eastern Mediterranean Region.

e EUR: WHO European Region.

f SEAR: WHO South-East Asia Region.

g WPR: WHO Western Pacific Region.

## Discussions

All reporting countries adapted, developed, and implemented information systems for monitoring COVID-19 vaccination; the approaches taken varied. For each of the 7 systems explored, nearly all responding countries reported that the system was in place for COVID-19 vaccines. Among these countries, for each system, at least half responded that the system was newly developed for COVID-19 vaccines. In total, 2 in 3 (n=127, 67.6%) countries reported establishing at least one new system, with around 72% of HIC or lower-middle income countries and around 61% of upper-middle income countries or LICs reporting this. Concurrently, many countries reported adapting existing systems to include COVID-19 vaccines, notably those for stock management and safety surveillance. Overall, 55.3% (n=104) of countries reported adapting at least one system already in place to COVID-19 vaccines, with 62% (n=36) 0f the HICs reported adapting at least one system already in place compared to around 53% for the other income groups. On digital health frameworks, 31% (n=46) of responding countries reported needing to adapt them in relation to COVID-19 vaccination systems, mostly HICs.

The present analysis demonstrates an unprecedented, widespread investment in IIS due to the COVID-19 vaccine rollout, and in particular in digital solutions. The unique data demands of the COVID-19 vaccine rollout favored the stand up of digital systems, as they can allow for the capture of more granular data and can make this information available to stakeholders quickly and directly. Further, development partners made considerable financing available to countries for such investments in digital systems. A recent analysis demonstrated that the World Bank, UNICEF, Gavi, and the Global Fund committed nearly US $9.5 billion for vaccine delivery projects containing at least one digital aspect [12]. The global immunization monitoring community recognized in March 2023, however, that nondigital systems too are able to meet COVID-19 vaccination monitoring recommendations, when appropriately implemented and resourced [13].

The benefits of investments made are already discernable in some countries. In Ethiopia and Pakistan, investments made in safety monitoring systems streamlined the collection, analysis, and reporting of adverse event following immunizations, allowing for more timely and comprehensive investigation of potential safety signals [14]. In England, development of their National Immunisations Management System enabled producing some of the world’s first, and more consistent vaccine effectiveness estimates over time, which was more difficult under the fragmented, noncentral systems of before [9]. In India, integration of existing systems with new ones and interoperability investments under their COVID-19 Vaccine Intelligence Network created an end-to-end data ecosystem, allowing for efficient service delivery and digital certificate deployment at scale [15]. To our knowledge, a full assessment of benefits has not yet been summarized, however.

Previous experience has demonstrated, and observations from the COVID-19 pandemic period confirm, that the implementation of new digital systems can be challenging in many contexts. Such systems require strong institutional foundations and significant material, financial, and human resources both at setup and during their operation. Since the period covered by this analysis, many countries have experienced difficulty in implementing or maintaining new digital systems for COVID-19 vaccine monitoring, limiting their impact. As an example, many countries opting to implement new e-trackers or EIRs for COVID-19 developed substantial data entry backlogs due to the time required for vaccination event recording and the sheer volume of entries to be made [16]. A rapid assessment of data systems in 23 African countries showed that in the 18 countries having adopted a new EIR for COVID-19, only 61% of COVID-19 vaccinations had been entered into the digital systems, on average [17]. As a result, most reverted at least partially back to the systems in prior use, creating duplicate reporting streams.

As COVID-19 vaccination programs evolve, and integrate into routine systems, reflection is needed on how to carry forward investments made in IIS during the pandemic period. Recognizing the evolution of COVID-19 programming, WHO and UNICEF now recommend integrating COVID-19 vaccination, including its monitoring and associated IIS, with immunization programmes and primary health care services [18]. For monitoring, countries need to plan how to do so based on their programmatic and fiscal contexts. In some, this will mean further expanding on systems developed during the COVID-19 vaccine rollout. England, Ghana, India, and Nigeria for example will expand their COVID-19-specific EIRs to include other routine vaccines [8,9,19,20]. This approach may not be suitable across all countries, especially those that had difficulties implementing new systems. At the time of survey, 47% (n=60) of countries that developed new systems reported they intended to expand at least one new system to other vaccines. This figure emphasizes the important need for renewed IIS development and sustainability planning.

The present analysis provides the first global snapshot of the systems used and the associated developmental approach taken to monitor COVID-19 vaccination. Covering the 2021 period, the present analysis provides an early data point during the vaccine rollout, representing both concrete actions taken that year as well as initial country intentions for the future. It serves as a useful benchmark against which system development can be gauged. Building on this, further investigation is now needed to understand: (1) how country approaches changed over time, (2) how COVID-19 vaccination IIS are being routinized across countries, and (3) under which circumstances IIS are being further built upon. The data presented here were self-reported via a questionnaire without validation rules, however, allowing for possible misreporting. Due to this design, it is not possible to discern which responses may be erroneous, especially in the case of double reporting when multiple answers may reflect responses for multiple health system levels. We also acknowledge, as well, that in such cases respondents may have been uncertain as to which response best reflected their national situations, for example regarding the legal framework. Further, being just a component of the overall supplemental questionnaire, which is itself a small piece of the overall JRF, the reporting burden on responding Ministry of Health staff is considerable. This burden is the main cause for nonreporting. Additional data are being collected through the 2023 JRF, administered in 2024, to address these future directions and the above-mentioned shortcomings.

As countries chart the way forward, continued support for and investment in IIS is critical. Not only is it important for continued immunization program improvement (notably for the identification and vaccination of zero dose and under-vaccinated children) and the achievement of national and global immunization goals, but also to ensure investments made during the COVID-19 period were not for naught [21]. Insufficient material and human resources have been highlighted repeatedly as key blockages to the successful implementation of new systems [17,22]. The COVID-19 vaccine rollout has further highlighted important foundational areas requiring concerted work, notably around data and digital health governance, system interoperability, and training of health staff with data-related functions. Countries and development partners must continue to prioritize strengthening these areas, even if they are long-term investments and typically nonpreferred partner investment areas, for better routine immunization services and preparedness for future epidemic or pandemic disease outbreaks [13].

## Supplementary material

10.2196/62657 Multimedia Appendix 1 Format and structure of the question included in the JRF supplemental questionnaire to assess the information systems used to manage COVID-19 vaccines per country. JRF: WHO/UNICEF (World Health Organization/United Nations Children's Emergency Fund) Joint Reporting Form on Immunization.

10.2196/62657 Multimedia Appendix 2 Tooltips and descriptions included as part of the information system question of the JRF supplemental questionnaire to support accurate country reporting. JRF: WHO/UNICEF (World Health Organization/United Nations Children's Emergency Fund) Joint Reporting Form on Immunization.

10.2196/62657 Multimedia Appendix 3 Analysis of country use of 7 information systems for the management of COVID-19 vaccination, at the global level and by country grouping.

10.2196/62657 Multimedia Appendix 4 Raw JRF supplemental questionnaire response and country characteristic data. JRF: WHO/UNICEF (World Health Organization/United Nations Children's Emergency Fund) Joint Reporting Form on Immunization.

## Abbreviations

eHBR: electronic home-based record
EIR: electronic immunization registry
HIC: high-income country
IIS: immunization information systems
JRF: World Health Organization/United Nations Children's Emergency Fund Joint Reporting Form on Immunization
LIC: low income country
UNICEF: United Nations Children's Emergency Fund
UNICEF: United Nations International Children's Emergency Fund
WHO: World Health Organization
