# Supplementary figures and images for: Developing National Information Systems to Monitor COVID-19 Vaccination: A Global Observational Study
Source: JMIR Public Health Surveill. 2024 Oct 25;10:e62657. doi: 10.2196/62657 (PMC11529800; doi:10.2196/62657)

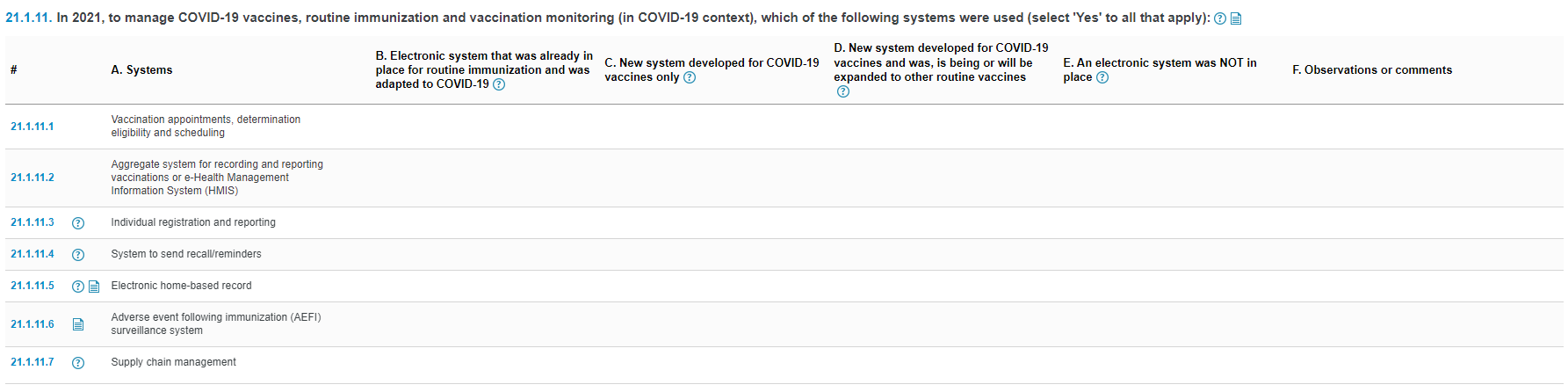

Supplement: Multimedia Appendix 1 [file publichealth-v10-e62657-s001.png]
