# Supplementary material for: Developing National Information Systems to Monitor COVID-19 Vaccination: A Global Observational Study
Source: JMIR Public Health Surveill. 2024 Oct 25;10:e62657. doi: 10.2196/62657 (PMC11529800; doi:10.2196/62657)
Supplement: Multimedia Appendix 2 [file publichealth-v10-e62657-s002.docx]

| Questionnaire text | Associated tooltip | Associated instruction |
| --- | --- | --- |
|  |  |  |
| In 2021, to manage COVID-19 vaccines, routine immunization and vaccination monitoring (in COVID-19 context), which of the following systems were used (select ‘Yes’ to all that apply): | Select ‘Yes’ to all that apply. More than one ‘Yes’ to each system is possible, particularly in decentralized countries, or overtime. | If more than one ‘Yes to each system is selected, particularly in decentralized countries, or overtime, please explain in the comment column. |
| Individual registration and reporting | Electronic immunization registry or e-tracker |  |
| System to send recall/reminders | Inform users when to go back to receive their next dose, or if remind delayed doses |  |
| Electronic home-based record | e-Vaccination Card, QR code | A home-based record is a health document – issued to an individual by an official authority, such as a national, provincial or district health department – on which an individual’s history of health services and/or vaccinations received from all healthcare providers is recorded. |
| Adverse event following immunization (AEFI) surveillance system |  | A national system must include ALL of the following:   1. Written guidelines on monitoring and investigation of reported adverse events; 2. A written list of events to monitor; 3. An established mechanism to communicate data for regulatory action; and 4. Implementation of points 1, 2 and 3. |
| Supply chain management | e-stock management system |  |
| Electronic system that was already in place for routine immunization and was adapted to COVID-19;  New system developed for COVID-19 vaccines only;  New system developed for COVID-19 vaccines and was, is being or will be expanded to other routine vaccines;  An electronic system was NOT in place | Select ‘Yes’ if this statement is correct |  |
