# Supplementary material for: Developing National Information Systems to Monitor COVID-19 Vaccination: A Global Observational Study
Source: JMIR Public Health Surveill. 2024 Oct 25;10:e62657. doi: 10.2196/62657 (PMC11529800; doi:10.2196/62657)
Supplement: Multimedia Appendix 3 [file publichealth-v10-e62657-s003.docx]

|  | Variable | Supply system  (N = 101) | EIR  (N = 116) | eHBR  (N = 79) | Aggregate reporting system  (N = 118) | Safety system  (N = 125) | Appointment system  (N=124) | Reminder system  (N = 87) |
| --- | --- | --- | --- | --- | --- | --- | --- | --- |
| **Overall (N=188)** | N of countries responding  (% of countries that submitted) | 101 (53.7) | 116 (61.7) | 79 (42) | 118 (62.8) | 125 (66.5) | 124 (66.0) | 87 (46) |
|  | N of countries that used the system  (% of those responding) | 98 (97) | 110 (94.8) | 71 (90) | 116 (98.3) | 116 (92.8) | 116 (93.5) | 77 (89) |
| **WHO region** |  |  |  |  |  |  |  |  |
| **AFR**  **(N=46)** | N of countries responding  (% of countries that submitted) | 27 (59) | 28 (61) | 18 (39) | 32 (70) | 34 (74) | 32 (70) | 21 (46) |
|  | N of countries that used the system  (% of those responding) | 26 (96) | 26 (93) | 16 (89) | 32 (100) | 32 (94) | 28 (88) | 17 (81) |
| **AMR**  **(N=35)** | N of countries responding  (% of countries that submitted) | 21 (60) | 22 (63) | 21 (60) | 23 (66) | 24 (69) | 22 (63) | 15 (43) |
|  | N of countries that used the system  (% of those responding) | 21 (100) | 21 (95) | 20 (95) | 21 (91) | 22 (92) | 21 (95) | 13 (87) |
| **EMR**  **(N=19)** | N of countries responding  (% of countries that submitted) | 9 (47) | 11 (58) | 5 (26) | 11 (58) | 9 (47) | 11 (58) | 8 (42) |
|  | N of countries that used the system  (% of those responding) | 9 (100) | 11 (100) | 4 (80) | 11 (100) | 9 (100) | 11 (100) | 8 (100) |
| **EUR**  **(N=53)** | N of countries responding  (% of countries that submitted) | 27 (51) | 33 (62) | 24 (45) | 30 (57) | 34 (64) | 35 (66) | 28 (53) |
|  | N of countries that used the system  (% of those responding) | 26 (96) | 30 (91) | 21 (88) | 30 (100) | 31 (91) | 33 (94) | 25 (89) |
| **SEAR**  **(N=11)** | N of countries responding  (% of countries that submitted) | 7 (64) | 8 (73) | 7 (64) | 9 (82) | 10 (91) | 9 (82) | 6 (55) |
|  | N of countries that used the system  (% of those responding) | 6 (86) | 8 (100) | 6 (86) | 9 (100) | 8 (80) | 8 (89) | 5 (83) |
| **WPR**  **(N=24)** | N of countries responding  (% of countries that submitted) | 10 (42) | 14 (58) | 4 (17) | 13 (54) | 14 (58) | 15 (63) | 9 (38) |
|  | N of countries that used the system  (% of those responding) | 10 (100) | 14 (100) | 4 (100) | 13 (100) | 14 (100) | 15 (100) | 9 (100) |
| **WB income classification** |  |  |  |  |  |  |  |  |
| **Low**  **(N=26)** | N of countries responding  (% of countries that submitted) | 12 (46) | 15 (58) | 10 (38) | 17 (65) | 17 (65) | 17 (65) | 12 (46) |
|  | N of countries that used the system  (% of those responding) | 12 (100) | 14 (93) | 7 (70) | 17 (100) | 16 (94) | 15 (88) | 10 (83) |
| **Lower-middle (N=51)** | N of countries responding  (% of countries that submitted) | 26 (51) | 32 (63) | 19 (37) | 35 (69) | 37 (73) | 35 (69) | 22 (43) |
|  | N of countries that used the system  (% of those responding) | 25 (96) | 30 (94) | 17 (89) | 35 (100) | 33 (89) | 32 (91) | 18 (82) |
| **Upper-middle**  **(N=51)** | N of countries responding  (% of countries that submitted) | 29 (57) | 33 (65) | 23 (45) | 29 (57) | 33 (65) | 31 (61) | 25 (49) |
|  | N of countries that used the system  (% of those responding) | 28 (97) | 30 (91) | 22 (96) | 27 (93) | 31 (94) | 28 (90) | 22 (88) |
| **High**  **(N=58)** | N of countries responding  (% of countries that submitted) | 34 (59) | 36 (62) | 27 (47) | 36 (62) | 37 (64) | 41 (71) | 28 (48) |
|  | N of countries that used the system  (% of those responding) | 33 (97) | 36 (100) | 25 (93) | 36 (100) | 36 (97) | 41 (100) | 27 (96) |
| **Gavi eligibility** |  |  |  |  |  |  |  |  |
| **Eligible**  **(N=57)** | N of countries responding  (% of countries that submitted) | 27 (47) | 34 (60) | 20 (35) | 40 (70) | 40 (70) | 36 (63) | 23 (40) |
|  | N of countries that used the system  (% of those responding) | 27 (100) | 31 (91) | 16 (80) | 40 (100) | 36 (90) | 31 (86) | 18 (78) |
| **Not eligible**  **(N=131)** | N of countries responding  (% of countries that submitted) | 74 (56) | 82 (63) | 59 (45) | 78 (60) | 85 (65) | 88 (67) | 64 (49) |
|  | N of countries that used the system  (% of those responding) | 71 (96) | 79 (96) | 55 (93) | 76 (97) | 80 (94) | 85 (97) | 59 (92) |
| **COVID coverage** |  |  |  |  |  |  |  |  |
| **<25%**  **(N=44)** | N of countries responding  (% of countries that submitted) | 22 (50) | 27 (61) | 15 (34) | 31 (70) | 31 (70) | 28 (64) | 17 (39) |
|  | N of countries that used the system  (% of those responding) | 22 (100) | 25 (93) | 13 (87) | 30 (97) | 30 (97) | 24 (86) | 13 (76) |
| **25-49%**  **(N=45)** | N of countries responding  (% of countries that submitted) | 26 (58) | 28 (62) | 18 (40) | 25 (56) | 29 (64) | 28 (62) | 23 (51) |
|  | N of countries that used the system  (% of those responding) | 25 (96) | 25 (89) | 15 (83) | 24 (96) | 24 (83) | 25 (89) | 18 (78) |
| **50-74%**  **(N=58)** | N of countries responding  (% of countries that submitted) | 29 (50) | 37 (64) | 27 (47) | 40 (69) | 39 (67) | 41 (71) | 28 (48) |
|  | N of countries that used the system  (% of those responding) | 28 (97) | 36 (97) | 25 (93) | 40 (100) | 36 (92) | 40 (98) | 27 (96) |
| **≥75%**  **(N=39)** | N of countries responding  (% of countries that submitted) | 23 (59) | 23 (59) | 18 (46) | 21 (54) | 25 (64) | 26 (67) | 18 (46) |
|  | N of countries that used the system  (% of those responding) | 22 (96) | 23 (100) | 18 (100) | 21 (100) | 25 (100) | 26 (100) | 18 (100) |
